# Concomitant Idiopathic Pulmonary Fibrosis and Lung Cancer: An Updated Narrative Review

**DOI:** 10.3390/arm93040031

**Published:** 2025-08-18

**Authors:** Bartłomiej Czyżak, Sebastian Majewski

**Affiliations:** Department of Pneumology, Medical University of Lodz, 90-153 Lodz, Poland

**Keywords:** idiopathic pulmonary fibrosis, lung cancer, combined pulmonary fibrosis and emphysema, acute exacerbation

## Abstract

**Highlights:**

**What are the main findings?**

- Lung cancer (LC) in patients with idiopathic pulmonary fibrosis (IPF) occurs more frequently compared to the general population.
- IPF and LC share common risk factors and overlapping pathobiological mechanisms.
- The coexistence of IPF and LC is particularly concerning, as it is associated with poor prognosis and a high risk of complications related to both diagnosis and treatment.

**What is the implication of the main finding?**

- The development of standardized management guidelines for patients with coexisting IPF and LC is essential to improve clinical outcomes.
- Novel therapeutic agents that simultaneously target the fibrotic and neoplastic pathways of IPF and LC are currently under investigation.

**Abstract:**

Idiopathic pulmonary fibrosis (IPF) is a chronic and progressive interstitial lung disease (ILD) with poor prognosis and limited therapeutic options. The introduction of antifibrotic agents has improved survival outcomes in IPF patients, which has led to more frequent recognition of comorbidities, particularly lung cancer (LC). This review summarizes current evidence on the epidemiology and pathogenesis of LC in the context of IPF, with particular emphasis placed on shared molecular, cellular, genetic, and epigenetic alterations. Diagnostic approaches and available treatment modalities, including surgical, systemic, and radiation therapies, are outlined, and their limitations in patients with IPF-LC are discussed. Acute exacerbations (AEs), as a life-threatening complication influencing diagnostic and treatment strategies, are specifically addressed. Moreover, studies indicating a possible protective effect of antifibrotic agents against LC development in IPF are reviewed. Further research is warranted into the shared mechanisms of IPF and LC to identify novel therapeutic targets. Establishing standardized, multidisciplinary clinical guidelines is essential for optimizing patient management, reducing AE risk, and improving patient outcomes.

## 1. Introduction

Idiopathic pulmonary fibrosis (IPF) is characterized by the progressive accumulation of excessive fibrosis in the lung parenchyma due to unknown causes. This leads to structural distortion of lung architecture, resulting in a usual interstitial pneumonia (UIP) pattern visible in radiological and histopathological examinations. Consequently, lung function deteriorates, physical capacity declines, and chronic respiratory failure develops, ultimately leading to premature death [1,2]. The pathogenesis of IPF is driven by chronic, repetitive subclinical micro-injuries to alveolar epithelial cells (AECs), triggered by smoking, air pollution, including traffic pollution and ambient pollution, viral infections, occupational exposures, and microaspirations [3,4,5]. Additional risk factors for IPF development include male sex and advanced age [1,5].

A frequent phenomenon is the coexistence of fibrosis and emphysema, which is termed combined pulmonary fibrosis and emphysema (CPFE) [6,7]. IPF is the most frequent fibrotic interstitial lung disease (fILD), and the UIP is the most common pattern in CPFE, but other types of pulmonary fibrosis can also be involved [7,8,9]. The definition of CPFE has evolved, making data comparisons between studies challenging. Currently, CPFE is categorized into research and clinical syndromes [7].

Before the advent of antifibrotic therapies, the median survival after an IPF diagnosis was approximately 3 to 5 years [10]. Nintedanib and pirfenidone, the standard-of-care drugs for IPF treatment, can only slow down the progression of fibrosis. Pivotal registration studies suggest that antifibrotic agents reduce the annual decline in forced vital capacity (FVC) by approximately 50% as compared with placebo [11,12,13]. Moreover, longitudinal real-world data confirm that both drugs attenuate lung function decline in population-based cohorts of patients with IPF [14,15]. Antifibrotic therapies lead to prolonged survival of IPF patients, causing the increased revelation of lethal comorbidities that would possibly not be noticed because of shorter survival in the era before antifibrotics, such as lung cancer (LC) [16].

**Figure 1:**
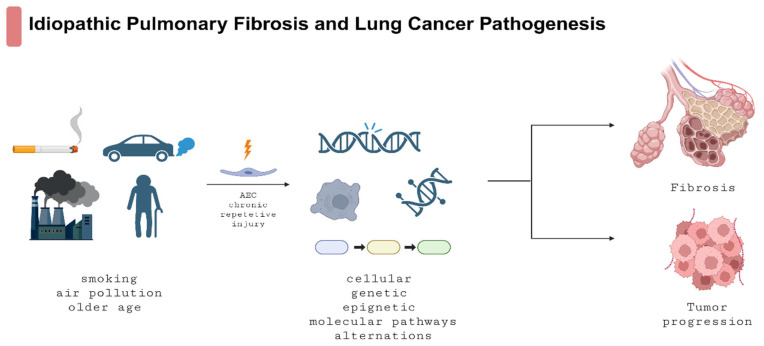
Idiopathic pulmonary fibrosis and lung cancer pathogenesis. Abbreviations: AEC—alveolar epithelial cell.

LC is the leading cause of new cancer cases and cancer-related deaths worldwide, with 2.5 million new diagnoses and 1.8 million deaths in 2020 [17]. As in IPF, progression in LC constitutes a significant and inherent feature of the disease’s natural history. IPF and CPFE significantly magnify the risk of its development. The prevalence of LC in the IPF and CPFE populations is several times higher than in the general population, and significantly worsens patients’ prognosis [18,19,20]. Smoking, air pollution, older age, and male sex contribute to both diseases, which can be explained by common molecular, cellular, genetic, and epigenetic alterations in IPF and LC pathogenesis [1,21,22,23]. Figure 1 illustrates the common pathogenesis of IPF and LC.

The diagnosis and management of LC in IPF patients pose significant challenges, often contributing to poor outcomes. Despite advances in research, much remains unknown about IPF-LC, and further studies are needed to optimize patient care. This narrative review aims to summarize current knowledge regarding the coexistence of IPF and LC, with a focus on epidemiology, pathogenesis, diagnosis, and treatment strategies.

## 2. Epidemiology

### 2.1. Epidemiology of LC in Patients with IPF

The risk of LC in IPF patients is significantly higher than in the general population, with IPF being recognized as an independent risk factor for LC development [18]. The reported LC incidence in IPF patients varies across studies, ranging from 3.3% to 20.5%, as shown in Table 1. The duration of IPF correlates with cancer risk, increasing progressively over time. Reported incidence rates range from 1.1% to 5.5% at 1 year, 4.7% to 11.4% at 3 years, 12.2% to 15.9% at 5 years, and 26.6% to 54.7% at 10 years [20,24,25,26,27]. IPF is usually diagnosed before LC, but in some cases, LC diagnosis precedes IPF, or both conditions are diagnosed simultaneously. In a large multicenter observational study from seven European countries by Karampitsakos et al., IPF and LC were diagnosed simultaneously in 32.7% of cases, while in 9.3% of cases, the diagnosis of LC preceded that of IPF. In other studies, these figures vary, ranging from 10% to 36.5% and from 5.9% to 32% [24,27,28,29,30]. Median time intervals between IPF and LC diagnosis range from 17.2 to 41 months, although one retrospective cohort study by Ozawa et al. reported a much longer interval of 120 months [Table 1]. Studies indicate conflicting results regarding LC’s impact on the survival of patients with IPF. Some studies declare that there is no relevant difference between survival in IPF-LC and IPF [25,28,31]. However, other studies report that LC in IPF patients significantly shortens survival. In a retrospective cohort study by Yoo et al., the median survival of patients with LC was 3.4 years compared to 9.8 years in those without LC. Furthermore, observational studies, including large multicenter studies, demonstrated that the presence of LC is associated with an increased risk of all-cause mortality in IPF patients [20,24,27,32].

### 2.2. Risk Factors for LC Development in IPF

Several factors increase the likelihood of LC development in IPF patients, including older age, male sex, smoking history, and emphysema [20,25,28,32]. E-cigarette vapor contains compounds with known carcinogenic potential in human cells. However, the association between vaping and the development of LC remains poorly established [33]. The relationship between vaping and ILD is even less understood. While there is currently no direct evidence linking e-cigarette vapor to the onset of ILD, several components of the vapor have been shown to induce pulmonary inflammation and oxidative stress. These processes may lead to the generation of reactive oxygen species (ROS), which could, in turn, influence fibroblast activity and promote fibrotic remodeling of lung tissue [34]. According to literature data, the median age at LC diagnosis ranges from 66.8 years to 71.38 years [Table 1]. An early IPF diagnosis is also a risk factor for LC development due to longer disease duration [25]. On average, men constitute 82.6–94.8% of IPF-LC patients [Table 1]. Kato et al. observed that a smoking history of ≥35 pack-years significantly increases the risk of LC development [28]. The median number of pack-years in IPF-LC patients according to different studies ranges between 36.7 and 73.1 [Table 1]. Notably, smoking is not an essential risk factor for the development of IPF and LC; however, never-smokers still constitute a fraction of IPF-LC patients compared to the group with a history of smoking. According to retrospective studies, never-smokers constitute around 5.9-14.2% of IPF-LC patients, and 2.72 to 4.2% of IPF patients develop LC [20,24,27,32,35,36].

Similarly to the general population, IPF patients with coexisting emphysema are more likely to develop LC compared to subjects without emphysema. Many observational studies did not include emphysema as a reported variable in the clinical characteristics of studied cohorts. However, pulmonary function tests (PFTs) in IPF-LC patients frequently show high FVC and total lung capacity (TLC) values, along with a lower forced expiratory volume in 1 second (FEV1) to FVC ratio (FEV1/FVC), suggesting a high prevalence of emphysema in this group [20,25,27,28,32,37]. Studies that reported emphysema coexistence found that 35.3% to 60% of IPF-LC patients had emphysema [27,28,29,30]. Due to previously inconclusive criteria for emphysema severity in CPFE, not all cases were classified as CPFE.

### 2.3. Epidemiology of LC Concomitant with CPFE

The incidence of LC in CPFE patients ranges from 8.1% to 26.9% [19,38,39]. Within the broader LC population, CPFE has a prevalence of 7.3% to 8.9% [40,41]. The risk of LC increases with CPFE duration, much like in IPF [38,39]. CPFE is associated with a greater risk of LC compared to emphysema without fibrosis [35]. Patients with CPFE-UIP tend to be heavier smokers than IPF patients, and they are also more often males [19,35,42,43,44]. The median number of pack-years in the CPFE-LC group oscillates between 40 and 54, with 93% to 100% of cases occurring in men [39,40,41,45,46,47]. Studies indicate that LC is more common in CPFE than in IPF alone. A recent systematic review and meta-analysis identified an increased odds ratio (OR) of 2.69 for LC development in CPFE-UIP patients compared to IPF patients. This could result from a synergistic effect between fibrosis and emphysema, or simply the sum of these risk factors [19]. The median survival time following CPFE diagnosis varies between 19.5 and 49 months [38,39,47]. CPFE-LC patients have shorter survival times than those with emphysema–LC or LC without emphysema [35,40,41,47]. While research has not shown significant survival differences between CPFE-LC and pulmonary fibrosis (PF) with LC, no studies have directly compared survival outcomes between CPFE-LC and IPF-LC [41]. Future research should fill this knowledge gap.

### 2.4. Localization and Histologic Type of LC in IPF

In IPF patients, non-small-cell lung cancer (NSCLC) is more prevalent (74.3–88%) than small-cell lung cancer (SCLC) (13–19%), which is consistent with the general population. The most common histologic type of LC in IPF is squamous cell carcinoma (SCC), which accounts for 33.3% to 39% of cases. This differs from the general population, where adenocarcinoma (ADC) is the most common variant, but not among smokers, where SQC is the most common histologic type, as in IPF patients [20,24,25,27,28,29,32,36,48,49]. According to Masai et al., in non-UIP patients, the predominant ADC subtype was papillary, whereas in UIP-ADC patients, it was mucinous [50,51]. Tumors in IPF-LC are primarily located in peripheral parts of the lungs (77–82.9%), in the lower lobes (48.6–58.7%), and mostly adjacent to or within fibrotic UIP lesions (74–77%) [20,24,28,29,32,48]. Similarly, in CPFE patients, tumors are adjacent to or within lung parenchymal changes (91.6–100%), localized peripherally (66.6–97.2%), and predominantly in the lower lobes (55.5–62%) [38,41,45,46,52]. Most of these parenchymal changes are fibrotic; however, in the study by Fujiwara et al., 54.3% of cases had emphysematous lesions compared to 42.9% with fibrotic lesions. Similarly to IPF, SCC is also the most common histologic type of LC in CPFE, accounting for 41.7–63.4% of cases [35,38,39,41,45,47,52]. Moreover, SCC has a higher prevalence, while ADC has a lower prevalence in CPFE than in the general population [47].

## 3. Common Cellular, Molecular, and Epigenetic Mechanisms in IPF and LC

### 3.1. The Role of Myofibroblasts and Other Cells in IPF Development

The main mechanism of IPF development is associated with the deposition of excessive fibrous tissue in the lung parenchyma, primarily driven by myofibroblasts. The fibrotic process begins with persistent insults to aging alveolar cells, leading to recurrent micro-injuries of the alveolar epithelium. Type II alveolar epithelial cells (AECs II) initiate impaired regenerative responses, and unsuccessful repair mechanisms trigger the release of pro-fibrotic cytokines, such as transforming growth factor-beta (TGF-β), which subsequently drive fibroblast differentiation into myofibroblasts and stimulate fibrous tissue deposition [23,53,54,55,56].

TGF-β further promotes fibrosis by inducing growth factors such as platelet-derived growth factor (PDGF), fibroblast growth factor (FGF), vascular endothelial growth factor (VEGF), and transforming growth factor-alpha (TGF-α). These factors contribute to fibroblast proliferation and angiogenesis, accelerating fibrosis [57,58,59]. Macrophages, particularly the M2 subtype, play a pivotal role in fibrosis by secreting profibrogenic cytokines and matrix metalloproteinases (MMPs) [23,60]. Unlike M1 macrophages, which mediate inflammation through cytokines like interleukin-6 (IL-6), interleukin-12 (IL-12), and tumor necrosis factor-alpha (TNF-α), M2 macrophages enhance tissue repair and fibrosis [61]. Lymphocytes also contribute to IPF pathogenesis, with Th2 and Th17 subpopulations playing key roles. Moreover, Th2 is a major subpopulation in IPF, which excretes interleukin 4 (IL-4), interleukin 5 (IL-5), interleukin 13 (IL-13), and TGF-β that are responsible for M2 macrophage recruitment and direct fibroblast activation [59]. Profibrogenic cytokines, growth factors, and MMPs are also excreted by senescent fibroblasts, called senescence-associated secretory phenotype (SASP) fibroblasts, which affect the microenvironment by altering gene expression of cytokines, growth factors, and proteases and inducing apoptosis resistance [23,50,54,62]. In IPF, SASP can be induced by the shortening of telomeres, for example, in the case of telomerase reverse transcriptase (*TERT*) or telomerase ribonucleic acid component (*TERC*) gene mutations, but also by environmental factors, which lead to oxidative stress, oncogene activation, deoxyribonucleic acid (DNA) damage, and chromatin abnormality. Furthermore, high concentrations of TGF-β suppress telomerase expression, accelerating telomere shortening and exacerbating fibrosis [62,63].

Activated myofibroblasts secrete extracellular matrix (ECM) components such as collagen type I, type III, and fibronectin while modifying collagen bundles through enzymatic activity to enhance fibrous tissue stability and resistance to degradation [56,64]. These cells establish strong adhesion via integrin-mediated focal adhesions and cadherin-mediated junctions, connecting their contractile cytoskeleton, which contains alpha-smooth muscle actin (α-SMA) [56,64,65]. Myofibroblasts in IPF primarily differentiate from residual interstitial fibroblasts; however, additional sources include pericytes, lipofibroblasts, mesothelial cells, and lung-resident mesenchymal stromal cells (LR-MSCs) [56,66,67,68,69]. Moreover, AECs II from epithelial-to-mesenchymal cell transition (EMT), vascular endothelial cells from the endothelial–mesenchymal transition (EndMT), and circulating fibrocytes that originate in bone marrow from mesenchymal progenitor cells are significant sources of myofibroblasts in IPF [23,56,70,71,72]. Research advancements enhancing our understanding of IPF pathobiology are crucial for developing novel therapeutic strategies and elucidating the connection between IPF and LC.

### 3.2. Cancer-Associated Fibroblasts (CAFs)

Beyond neoplastic cells, tumors consist of tumor stroma, which includes blood vessels and connective tissue. Tumor stroma nourishes neoplastic cells and facilitates cancer development. The fibroblast analogs in tumor stroma are cancer-associated fibroblasts (CAFs), which are α-SMA-positive myofibroblasts [56]. Interestingly, at early stages of cancer development, TGF-β delays tumorigenesis because it promotes apoptosis. However, once tumors have formed, TGF-β promotes progression by inducing CAF development [23,57,73,74,75]. CAFs arise through similar mechanisms to myofibroblasts in IPF, including EMT and EndMT, driven by cytokines such as TGF-β, epidermal growth factor (EGF), VEGF, FGF, TNF-α, interleukin 1β (IL-1β), and IL-6 secreted by cancer and non-malignant stromal cells [23,56,76]. Tumor stroma produced by CAFs enhances carcinogenesis by supporting tumor growth, metastasis, and drug resistance [56]. Tumor-associated macrophages are similar to M2 macrophages, which promote tumor growth by the activation of CAFs’ suppression of T-cell response [23,77,78]. CAFs contribute to ECM production, leading to the development of a stiff tumor stroma that disrupts blood vessel function. As a consequence, impaired vascularization reduces drug uptake, resulting in a poorer response to treatment [56,79,80]. Fibrotic changes in lung tissue not only increase stiffness but also enhance mechanical stretching. This impairs gas exchange, leading to a decline in lung function and contributing to the progression of both IPF and LC. Mechanosignaling is mediated through ECM–cell focal adhesions, which primarily consist of integrins, and also by growth factors released in response to mechanical forces. These mechanisms enable the transmission of signals to the nucleus by transductor proteins like focal adhesion kinase (FAK), phosphoinositide 3-kinase (PI3K), rat sarcoma virus oncogene (RAS), RAS homolog family member A (RhoA), and yes-associated protein (YAP), ultimately affecting signaling pathways that promote carcinogenesis and fibrosis, which is called the mechanistic link. Stiffness and mechanical stretch have been implicated in the initiation and progression of lung cancer from the perspective of proliferation, metastasis, angiogenesis, cancer stem cells (CSCs), immunology, epigenetics, and metabolism, which represent key biological processes involved in cancer development [56,81].

**Figure 2:**
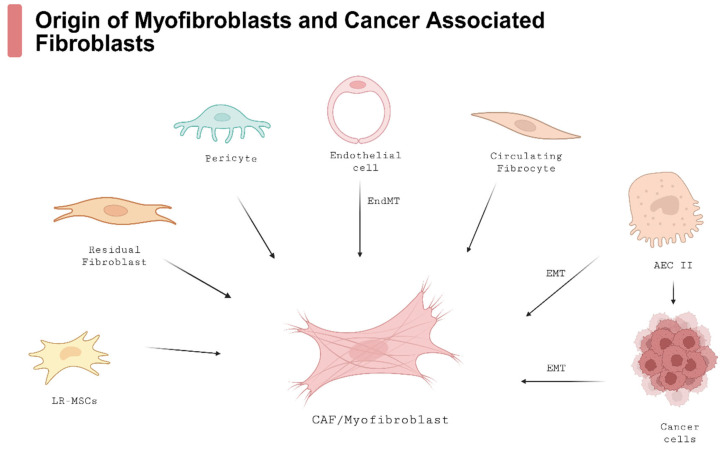
Origin of fibroblasts and cancer-associated fibroblasts. Abbreviations: LR-MSCs—lung-resident mesenchymal stromal cells; AEC II—alveolar epithelial cell type II; EndMT—endothelial–mesenchymal transition; EMT—epithelial–mesenchymal transition; CAF—cancer-associated fibroblast.

There is a hypothesis that CAFs, like myofibroblasts in IPF, gain the SASP. CAFs with the SASP excrete relevant growth factors, cytokines, and MMPs that promote tumor progression. However, cellular senescence may also act as a tumor-suppressive mechanism by limiting cancer cell proliferation [23,82,83]. Given the similar pathophysiology of CAFs and myofibroblasts in IPF, drugs developed for IPF could benefit anti-neoplastic therapies, and vice versa. Figure 2 summarizes the origins of myofibroblasts and CAFs in IPF and LC.

### 3.3. Tissue Invasion and Cell–Cell Communication

One of the hallmark features of cancer is its ability to invade local tissues, a process that also occurs in IPF. Myofibroblasts in IPF degrade basement membranes via MMPs, enabling fibrosis to spread; however, unlike cancer cells, they do not metastasize. The invasive potential of cancer cells is associated with molecules such as laminin, heat shock protein 27, and fascin [23,59,84,85,86]. Notably, overexpression of these proteins has been observed in IPF around fibroblast foci in bronchial basal cells, which constitute a layer between luminal epithelia and myofibroblasts. It enhances myofibroblast invasiveness and fibrosis propagation [59,87].

Connexin 43 (Cx43), a member of the connexin family, forms intracellular channels that synchronize cell proliferation and tissue repair [59,88]. Increased levels of TGF-β and excessive collagen production in IPF result in Cx43 downregulation, leading to loss of proliferative control, abnormal tissue repair, and fibrosis. Similarly, in LC, reduced Cx43 expression has been observed, and low connexin levels are often associated with cancer progression [23,59,89,90]. Integrins, another class of cell–cell communication molecules, regulate fibrosis in IPF and promote independent growth and drug resistance in cancer. In IPF, integrins are responsible for the initiation, maintenance, and resolution of fibrosis, so they are considered regulators of TGF-β. However, cancer cells, due to integrin activity, gain the potential for independent growth and drug resistance [59,91].

### 3.4. Endoplasmic Reticulum Stress and Unfolded Protein Response

Endoplasmic reticulum (ER) stress arises from the accumulation of misfolded proteins. One of the primary mechanisms for restoring homeostasis is the unfolded protein response (UPR), which regulates gene expression to enhance autophagy and degrade defective molecules. If this process fails, cells undergo apoptosis [23,56,59,92]. In IPF, autophagy is defective, leading to accelerated cellular senescence, ECM production, and resistance to apoptosis [23,62]. Furthermore, UPR activation stimulates the expression of pro-fibrotic mediators and EMT, exacerbating fibrosis [23,59,93,94]. In addition, ER stress may attenuate senescence and promote tumorigenesis. While autophagy may suppress carcinogenesis, in many cases, it facilitates tumor progression. Cancer cells often upregulate autophagy to withstand microenvironmental stress, thereby enhancing their survival, proliferation, and aggressiveness [23,95].

### 3.5. Molecular Pathways

A single mutation or alteration in gene expression can trigger molecular pathways that either initiate or advance disease progression. Several deregulated molecular pathways are common to both IPF and LC.

One such pathway is the PI3K/protein kinase B (AKT)/mammalian target of rapamycin (mTOR)-dependent pathway, which regulates proliferation and apoptosis. In IPF, the class I isoform p110γ, a component of the PI3K complex, is overexpressed [23,96]. This leads to the activation of downstream signaling of pro-fibrotic growth factors such as PDGF and TGF-β, promoting fibroblast proliferation and differentiation [23,97,98]. De-regulation of PI3K/AKT/mTOR in NSCLC has been linked to disease progression and the development of high-grade tumors due to uncontrolled proliferation and resistance to apoptosis [23,99].

Embryonic signaling pathways, including Wingless/Int beta-catenin (Wnt/β-catenin), Sonic Hedgehog (SHH), and Notch, are reactivated and overexpressed in both IPF and LC [23]. Wnt/β-katein regulates the expression of molecules that are involved in tissue invasion and tumor progression, such as matrilysin, laminin, and cyclin-D1. Additionally, the Wnt/β-katein pathway exhibits TGF-β crosstalk, promotes EMT and myofibroblast activation, and potentially increases the senescence of AECII [23,56,59,100].

The SHH pathway is reactivated in epithelial cells within honeycomb cysts and cancer stem cells. Its abnormal activation contributes to fibroblast resistance to apoptosis and facilitates tumor growth, metastasis, and chemotherapy resistance through paracrine signaling [56,101]. The reactivated Notch signaling pathway in AECs enhances EMT and α-SMA expression in fibroblasts. Notch signaling is frequently overactive, supporting cancer cell proliferation and inhibiting apoptosis [23,102,103]. Taken together, a detailed understanding of dysregulated molecular pathways in IPF-LC may aid in the development of novel therapeutic strategies. Identifying primary pathological mechanisms or indirectly affected molecules could help pinpoint new drug targets, ultimately leading to more effective treatments.

### 3.6. Germinal Mutations

Germinal mutations are responsible for the transition of pathological genes to the next generations, which may lead to familial disease clustering. Some of the germline mutations have been found in families with clustered interstitial pneumonia. Data suggest that in addition to the higher risk of developing IPF, these mutations also predispose to LC [23]. Among such is a germinal mutation of the pulmonary surfactant-associated protein A1 (*SPFTA1*) and A2 genes. Defective surfactant proteins accumulate in AECs II, triggering ER stress, which may contribute to IPF and cancer development [23,104,105,106]. Additionally, germinal *TERT* (rs2736100) and cyclin-dependent kinase inhibitor 1A (*CDKN1A*) (rs2395655) mutations, which impair telomerase function and DNA damage response, have been found in IPF families [23,107,108,109]. As previously mentioned, those mutations affect telomere shortening and lead to cells gaining SASP [62]. The same *TERT* (rs2736100) germline mutation has been identified as a risk factor for ADC, making it one of the most frequently altered genes in early-stage NSCLC [23,110,111]. Moreover, polymorphism in telomere-related genes *TERT*, *TERC*, the oligonucleotide/oligosaccharide-binding fold containing one gene (*OBFC1*), and regulator of telomere elongation helicase 1 (*RTEL1*) are associated with LC development by shortening of telomeres, leading to genomic instability [23,56,112,113,114,115]. A somatic and germline variant (rs35705950) in the promoter of the mucin 5B (*MUC5B*) gene is one of the risk factors for sporadic IPF and familial IPF development [23,116]. This polymorphism causes overexpression of MUC5B protein, which impairs mucociliary clearance and disrupts alveolar homeostasis [56,117]. This leads to disturbance in the AEC II repair processes and eventually, IPF development. Moreover, MUC5B gene variants have been associated with increased metastasis risk and poorer prognosis in NSCLC [23,56,118,119].

### 3.7. Somatic Mutations

The primary driver of LC development is the accumulation of mutations, particularly in tumor suppressor genes and oncogenes, many of which are also found in fibrotic tissue in IPF patients. A key example is the tumor suppressor gene, tumor protein 53 (*TP53*), which is frequently mutated in squamous metaplasia within IPF fibrotic tissue [23,56]. Other mutations affecting apoptosis regulation include p16, p21, and Kirsten rat sarcoma virus gene (*KRAS*), all detected in both IPF and LC [56,120]. Notably, IPF-LC patients exhibit lower frequencies of epidermal growth factor receptor (*EGFR*) and anaplastic lymphoma kinase (*ALK*) mutations compared to LC patients, impacting treatment options and prognosis [56,121]. Additional mutations include those in the fragile histidine triad (*FHIT*), which is a candidate tumor suppressor gene with functions that are not fully understood [23,122]. Loss of FHIT molecule function has been observed in NSCLC and IPF, with a higher frequency in IPF-LC cases than in IPF alone [23,123,124]. Likewise, mutations in the B-Raf proto-oncogene (*BRAF*), which encodes a serine/threonine kinase, have been identified in NSCLC and IPF-LC. BRAF inhibitors are used in LC treatment, but their efficacy is limited due to the rarity of the p.V600E mutation in IPF-LC [23,109]. Beyond oncogenes and tumor suppressors, immune-related genes like programmed cell death-ligand 1 (PD-L1) are dysregulated in both IPF and LC, which, after linking to the programmed cell death-1 (PD-1) molecule on lymphocytes, causes immune suppression, further promoting disease progression [125]. While there are significant similarities between IPF and LC pathogenesis, a key question remains: why does IPF drive LC development, but not vice versa? Besides mechanosignaling, which promotes carcinogenesis through stretching forces induced by fibrotic tissue, there is another theory that may be explained by differences in carcinogenesis between IPF-associated LC and LC without IPF. The most common mutation type in LC is the C > A transversion, which is strongly associated with a history of heavy smoking. However, in IPF-LC, C > T transitions predominate, despite patients often having a significant smoking history. This shift may be linked to the activity of the apolipoprotein B mRNA editing enzyme, catalytic polypeptide (APOBEC)—a cytidine deaminase that converts cytosine to uracil. Overexpression of APOBEC cytidine deaminase has been observed in tumor samples from IPF-LC patients. Research suggests that APOBEC activity may be triggered by viral infections or immune-related mutagenic processes, potentially serving as key drivers of LC development in the context of IPF [23,81,109].

### 3.8. Epigenetics

In addition to cellular and genetic abnormalities, a number of epigenetic factors contribute to the development of IPF and LC. Risk factors such as smoking, aging, and environmental pollution can trigger epigenetic responses, including gene methylation and abnormal micro-ribonucleic acid (miRNA) expression, which may be shared or interconnected in both diseases [23,56]. One of these responses is hypermethylation of the suppressor gene that encodes the SMAD family member 4 (SMAD4) protein, which is a signal transducer in the antiproliferative pathway that starts with TGF-β stimulation. In IPF, the overexpression of TGF-β leads to high SMAD4 protein concentrations in lung tissue. To compensate for excessive stimulation of this pathway, cells reduce SMAD4 gene expression through hypermethylation. As a result, despite high levels of TGF-β, SMAD4 concentration decreases, ultimately inhibiting the antiproliferative pathway [23,126,127]. Lack of proliferation inhibition may lead to carcinogenesis.

Moreover, O-6-methylguanine-DNA methyltransferase (MGMT) is a DNA repair enzyme that regulates chromatin stability and susceptibility to apoptosis [128]. The promoter of MGMT is one of the most hypomethylated genes in IPF; by contrast, hypermethylation of its promoter is one of the early events in carcinogenesis [23,128,129,130]. Furthermore, in NSCLC, MGMT hypermethylation occurs more frequently in smokers than in non-smokers [129]. However, no data regarding MGMT methylation in patients with IPF and LC are currently available.

On the other hand, hypermethylation of the thymus cell antigen 1 (*Thy-1*) gene promoter occurs in IPF, facilitating fibroblast differentiation into myofibroblasts [23,131]. In cancer, lack of *Thy-1* expression is associated with a more invasive course of the disease [23,130]. Studies confirm that around 10% of miRNAs are abnormally expressed in patients with IPF. Similarly, in LC, there are some miRNA expression disruptions affecting carcinogenesis. These molecules are involved in fibrosis, tumor growth, invasion, and metastasis by triggering fibrosis progression, regulation of ECM, EMT induction, and apoptosis. Similarly, in LC, some miRNA expression abnormalities affect carcinogenesis. Common miRNA abnormalities in both IPF and LC include decreased levels of lethal-7d (Let-7d), microRNA-29 (miR-29), and microRNA-200 (miR-200), alongside elevated levels of microRNA-21 (miR-21) and microRNA-155 (miR-155). However, data on the expression of these molecules in IPF-LC patients remain unavailable [23,59,130,132,133].

Research on cellular and molecular factors alone is insufficient to fully understand the complex relationship between IPF and LC. Epigenetics is bridging the knowledge gap regarding the coexistence of these diseases and the influence of shared environmental factors on their development.

### 3.9. Knowledge Gaps and Future Treatment Options

Many of the common cellular, molecular, and epigenetic factors discussed above in relation to IPF and LC still require further investigation and validation. A deeper understanding of these pathobiological mechanisms will facilitate the development of targeted therapies capable of halting fibrosis and tumor progression. Research in this area is already underway.

Nintedanib is a tyrosine kinase inhibitor that targets platelet-derived growth factor receptor (PDGFR), vascular endothelium growth factor receptor (VEGFR), and fibroblast growth factor receptor (FGFR). Notably, it is the first drug approved for the treatment of both diseases, serving as an antifibrotic agent in IPF and an anti-neoplastic drug in second-line therapy with docetaxel for ADC-NSCLC [23,134].

Pirfenidone is an approved antifibrotic treatment for IPF, and there is suspicion that pirfenidone also has anti-neoplastic potential. Some large retrospective studies support this statement, which will be discussed in more detail in the different paragraphs of this review [11,12,23,135,136]. Preclinical models suggest that combining pirfenidone with cisplatin may induce CAF and tumor cell death in NSCLC [23,137].

Among the most advanced novel therapies, research focuses on anti-TGF-β agents (Fresolimumab) and angiostatic compounds (Tetrathiomolybdate). Phase I/II trials have been completed on Tetrathiomolybdate in IPF therapy (NCT00189176), and the phase I study in LC treatment is recruiting patients (NCT01837329). Similarly, a phase I trial on Fresolimumab in IPF has been completed (NCT00125385), and in LC, this drug is in phase I/II (NCT02581787) [23].

Recent advancements in bioinformatics and biopharmaceutical technology have significantly enhanced the search for novel targeted therapies. Many studies have already leveraged these technologies to investigate IPF and LC. For instance, Dasgupta et al. took bronchoalveolar lavage fluid (BALf) and analyzed gene expression in LC, IPF, and control groups using the Gene Expression Omnibus to identify overlapping gene signatures. [138]. Their findings revealed 10 common genes: C-C motif chemokine ligand 13 (*CCL13*), C-X-C motif chemokine ligand 2 (*CXCL2*), mucosa-associated lymphoid tissue lymphoma translocation protein 1 (*MALT1*), myristoylated alanine-rich C-kinase substrate (*MARCKS*), phospholipase A2 group VII (*PLA2G7*), semaphorin 6B (*SEMA6B*), surfactant protein B (*SFTPB*), secreted protein acidic and cysteine rich (*SPARC*), secreted phosphoprotein 1 (*SPP1*), and TLC domain containing 2 (*TLCD2*), with phospholipase A2 group VII (*PLA2G7*) emerging as the most promising therapeutic target. High expression of *PLA2G7* was significantly associated with poorer survival of LC patients. In biopharmaceutical applications, darapladib has been identified as a potential PLA2G7 inhibitor, raising the possibility that it could serve as a novel therapeutic agent for fibrosis and tumor suppression [138]. However, further in vivo and in vitro studies are necessary to confirm its effectiveness. While the growing number of studies on targeted therapies for IPF and LC is promising, most remain in the early stages of research, highlighting the need for continued efforts and further validation.

## 4. Diagnosis of LC in IPF Patients

### 4.1. Imaging and Management of Suspected Malignancy

**Figure 3:**
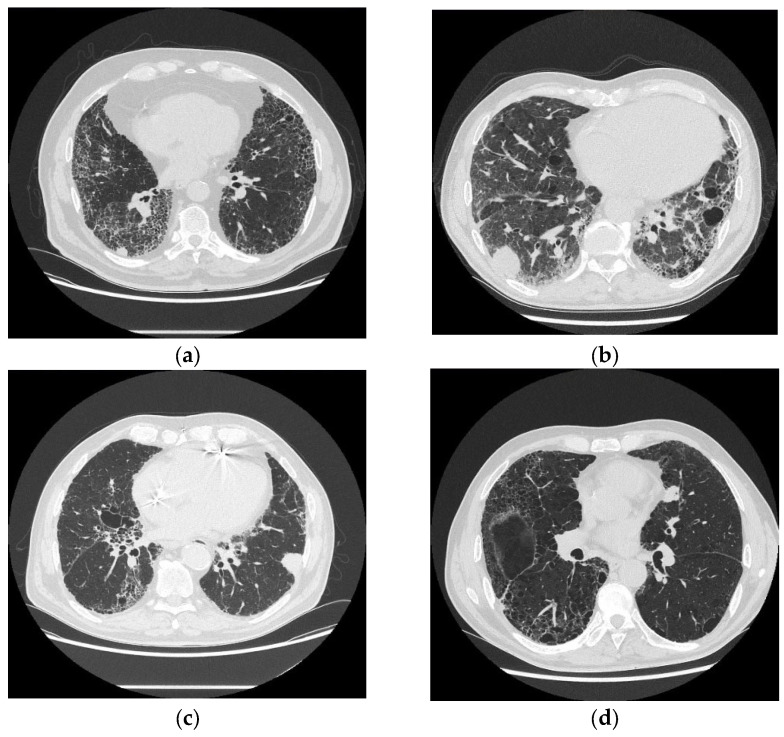
Examples of LC in patients with IPF on HRCT imaging. Panels (**a**–**c**): HRCT of the IPF-LC patients. Panel (**d**): HRCT of the CPFE-LC patient.

Medical imaging plays a crucial role in monitoring fibrosis progression in IPF patients. Additionally, regular high-resolution computed tomography (HRCT) assessments, commonly used for this purpose, may serve as a valuable screening tool for detecting LC at an early stage during patients’ follow-up [139,140]. LC developing in IPF is localized mostly adjacent to or within UIP regions (see Figure 3 with examples), making it challenging to differentiate dense fibrotic areas from malignant lesions [140,141]. Invasive mucinous ADC, for example, may present on computed tomography (CT) scans as a ground-glass opacity (GGO) with consolidative areas, potentially mimicking pneumonic lesions [140,142]. Furthermore, in IPF patients, assessment of mediastinal lymph nodes in the LC diagnostic process can be misleading. Enlarged mediastinal lymph nodes may occur in both IPF and LC, which reduces the specificity of their evaluation in the LC diagnostic process [50,143]. All those features significantly handicap imaging diagnostics of LC in IPF patients; therefore, any suspected new imaging findings in these patients should be considered a potential malignancy and require a thorough differential diagnosis. In clinical practice, nodules < 8 mm require repeated imaging after 3-6 months; if there is a progression on follow-up, in the next step, a positron emission tomography (PET-CT) scan is recommended. For 8 mm nodules, a PET-CT scan is highly recommended. If the PET-CT scan shows a significant uptake of fluorodeoxyglucose, a biopsy should be performed using minimally invasive procedures. In cases where a biopsy is not feasible or if the nodule is larger than 8 mm, a multidisciplinary and personalized approach should be considered [30,139,144].

### 4.2. Risks Related to Invasive Diagnostic Procedures of Suspected LC in Patients with IPF

For the complete differential diagnostic process of LC invasive procedures, such as bronchoscopy with or without bronchoalveolar lavage (BAL), transbronchial lung biopsy (TBLB), transbronchial lung cryobiopsy (TBLC), CT-guided transthoracic lung biopsy, or surgical lung biopsy (SLB), are typically required. Pneumothorax is one of the complications of CT-guided transthoracic lung biopsy that significantly increases mortality in IPF patients [50,145]. The presence of emphysema and honeycombing along the biopsy needle path is a key risk factor for pneumothorax, making IPF patients particularly susceptible due to these preexisting lung abnormalities [50,146,147]. Furthermore, fibrotic tissue surrounding the suspected lesion increases the risk that a biopsy would give a non-diagnostic result [50,146]. Beyond the standard risks of invasive procedures, acute exacerbation (AE) of the underlying interstitial lung disease (ILD) is a severe and potentially fatal complication [148]. AE-IPF refers to an acute worsening or development of dyspnea, typically developing within 30 days. It can be classified as triggered (due to infection, aspiration, drug toxicity, or invasive procedures) or idiopathic. According to the AE-IPF definition, lung imaging in AE-IPF reveals bilateral GGO and consolidations superimposed on a background of reticular or honeycomb lesions consistent with the radiologic UIP pattern. Clinically, AE-IPF must be distinguished from other acute events such as infection, PE, pneumothorax, and heart failure, all of which can manifest similarly. To relate AE-IPF with an identifiable trigger, such as surgery, AE must appear within 30 days after the procedure [149].

AE-IPF is a life-threatening condition that often requires intensive care unit (ICU) hospitalization. In severe cases, it leads to respiratory failure and death. Prognosis remains poor, with an in-hospital mortality rate of approximately 50%, and a median survival of just 3–4 months following an AE episode [148,149,150,151]. Additionally, even patients who survive an AE tend to have a worse long-term prognosis compared to those without a history of AE. A study by Song et al. found that IPF patients with a history of AE had a 5-year survival rate of only 18.4%, compared to 50% in those without AE-IPF [152]. The reported incidence of AE following SLB in ILD patients ranges between 1.5% and 7.4%, although procedures performed via video-assisted thoracoscopic surgery (VATS) have a lower AE risk of 1.05–2.22%. Patients who developed AE were older and had a lower vital capacity (VC), lower diffusing capacity of the lung for carbon monoxide (DLCO) before biopsy, and longer duration times of surgery and anesthesia [148,153,154,155]. AE has also been reported after TBLC, despite its less invasive nature compared to SLB. In Dhooria et al.’s study, the AE-ILD rate after TBLC was 2.34%; in the group of 128 patients, 3 patients developed AE, and all cases were fatal. Notably, severe bleeding and pneumothorax preceded AE in these cases, making it unclear whether AE was triggered by TBLC alone or as a complication of the preceding events [148,156]. Similar confounders and limitations were present in studies that observed a 1.99% to 2.42% incidence of AE after BAL, whereas a regular bronchoscopy and TBLB preceded BAL [148,157,158].

Invasive diagnostic procedures for suspected LC in IPF patients carry a significant risk of AE, necessitating careful selection of diagnostic modalities. Additionally, for some patients with suspected malignancy, the severity of fibrosis, advanced age, or comorbidities may render them ineligible for cancer treatment. In such cases, it may be more appropriate to refrain from invasive diagnostics and instead provide optimal supportive care. Nevertheless, real-world data on how often such a situation is seen in patients with IPF is missing.

## 5. Surgical Treatment of IPF-LC Patients

### 5.1. AE-IPF Risk in LC Resection Surgery

Resection surgery is a primary treatment for NSCLC, offering a potential cure. It is also utilized in IPF-LC cases; however, due to its greater invasiveness compared to diagnostic procedures, resection surgery carries a significantly higher risk of AE development [148]. According to available data, the reported incidence of AE in ILD patients after surgery varies widely from 4.9% to 30%, with high mortality rates of 12.50% to 83.3% (Table 2). The occurrence of postoperative AE-IPF is associated with a poor prognosis. Miyajima et al. showed that 5-year survival after NSCLC resection surgery complicated with postoperative AE was 12.2% compared to 43.3% in patients without AE history after surgery [150]. A multicenter retrospective cohort study by Sato et al., which included 1763 patients with NSCLC-ILD who underwent pulmonary resection surgery, found that 9.3% (n = 164) of patients developed postoperative AE with a mortality rate of 43.9%. The majority of postoperative AE events occurred within 10 days following surgery, with a median onset time of 7 days. The median time from AE onset to death was 20 days (range: 1–82 days). The 30-day mortality in the entire cohort of patients was 2.6%, although AE was responsible for 71% of deaths during this period. Wedge resection had the lowest risk of postoperative AE (3.6%), in contrast to segmentectomy/lobectomy (10%) and bilobectomy/pneumonectomy (16%). In comparison to wedge resection, segmentectomy (OR = 3.675 (95% CI, 1.586-8.519; *p* = 0.0024)) and lobectomy (OR = 3.861 (95% confidence interval [CI], 1.946-7.660; *p* < 0.001)) had a significantly higher risk of postoperative AE [159]. Available meta-analyses confirmed that sublobar resections are significantly associated with a lower risk of postoperative AE development [160,161]. Additionally, studies indicate that VATS does not reduce the risk of postoperative AE or 90-day postoperative mortality compared to thoracotomy [150,159,160,162].

### 5.2. Risk Factors of Postoperative AE

Besides the extension of surgery, many factors have been identified as increasing the risk of postoperative AE in ILD patients. Several studies using univariable logistic regressions have explored potential risk factors for postoperative AEs, including male gender, previous history of AEs, preoperative steroid use, levels of Krebs von den Lungen-6 (KL-6), percentage of predicted vital capacity (%VC), FEV1, DLCO, operative time, blood loss, CT findings, and type of surgical procedure [159,173]. However, in a multivariable logistic regression model, the identified risk factors were narrowed down to male gender, preoperative steroid use, level of KL-6, UIP pattern on CT, and low %VC predicted [159,173]. Additionally, two retrospective studies have suggested that high serum C-reactive protein (CRP) levels, body mass index (BMI), and a higher American Society of Anesthesiology (ASA) classification score may also contribute to an increased risk of AE [162,174]. Furthermore, Miyajima et al. found that male sex, VC ≤ 80%, neoadjuvant radiotherapy, AEs in the medical history, preoperative steroid therapy, type of surgical procedure, and emphysema were associated with a greater risk of death due to postoperative AEs [150]. Given these risk factors, careful patient selection, surgical planning, and postoperative management are essential to improving outcomes in ILD patients undergoing lung cancer resection

### 5.3. Overall Survival After Surgery in IPF-LC

Patients with IPF-LC have a lower overall survival (OS) following surgeries compared to LC accompanied by other types of ILDs [174]. The presence of a UIP pattern in CT scans is also associated with shorter survival. Factors that significantly influence OS in ILD patients at various stages of NSCLC after surgical treatment include DLCO, older age, the occurrence of AE after surgical treatment, stage of LC, and low %VC (<80% predicted in the IA stage) [169,170,174,175]. The Gender Age and Physiology (GAP) index, originally developed to provide a simple method to determine the average risk of mortality of IPF patients, may serve as a prognostic tool for predicting outcomes after LC resection in ILD patients. The ILD-GAP index is based on the type of ILD, sex, age, FVC, and DLCO, and it estimates 1, 2, and 3-year mortality in ILD patients. In Ueno et al.’s study, higher values of the ILD-GAP index were associated with increased risk of death after LC resection surgery; therefore, this index can serve as a prognostic tool in surgically resected patients with ILD and concomitant LC [174]. Data on OS after different types of LC resection procedures in ILD patients remain inconsistent. Studies examining ILD-LC in stages I-III have found no significant difference in OS between sublobar and lobar resection [161,169,170,172]. However, Sato et al. showed that OS in IA stage patients after wedge resection surgery was significantly lower than after lobectomy. In a large cohort of ILD patients who underwent LC resection, the leading cause of death was LC (50.2%), followed by respiratory failure due to AE-ILD (26.8%) [175]. Notably, wedge and segmental resection surgeries are associated with a higher frequency of LC-related deaths compared to lobectomies. This may be attributed to the lower radicality of these procedures and an increased risk of LC recurrence [175]. However, on the other hand, Patel et al. suggest that in highly selected cases with small tumor sizes, after a proper counseling process, wedge resection may be the best treatment option with similar outcomes to lobectomy [161]. Therefore, the selection of appropriate surgical procedures should be individualized. Such an approach may be challenging; hence, some guidelines and validated assessment scales are necessary to facilitate this process. Furthermore, current data regarding differences in the extension of lung resection in IPF-LC patients are derived from retrospective studies, in which the quality of evidence is insufficient due to limitations and possible confounding. Therefore, randomized controlled trials and prospective studies are required. Currently, a phase III study (JCOG1708, SURPRISE) is underway to evaluate survival outcomes, comparing the effectiveness of sublobar and lobar resections in resectable stage I NSCLC in IPF patients [176]

### 5.4. Prevention of Postoperative AE-IPF

There is currently no validated and effective method for preventing postoperative AE. However, antifibrotic therapy is considered the most promising approach for perioperative AE prevention, as it is suspected to help reduce the risk of postoperative AE development [173].

In a retrospective study, Iwata et al. evaluated 50 patients with IPF-LC who underwent LC resection surgery: 31 patients received pirfenidone, while 19 did not receive any antifibrotic treatment. The incidence of AE-IPF within 30 days postoperatively was 0% in the pirfenidone group and 10.5% in the non-antifibrotic group (*p* = 0.07) [177].

A more recent, multicenter Italian retrospective study included 55 patients with IPF-LC after LC resection surgery, 29 of whom used pirfenidone chronically or in the perioperative period. The incidence of postoperative AE in the pirfenidone group was significantly lower than in the non-antifibrotic group (3.4% vs. 23.1%, *p* = 0.044). However, there was no significant difference in mortality between these groups [178].

In the phase II trial by Iwata et al., 36 IPF-LC patients were treated with pirfenidone and underwent LC resection surgery; only 1 patient experienced AE (2.8%) [179]. However, the study had a small sample size and lacked a control group. Consequently, a large-scale phase III trial is currently underway [180]. Furthermore, the effectiveness and safety of perioperative nintedanib administration remain uncertain. The drug’s label does not recommend its use in the perioperative period due to potential interference with the wound-healing process [173].

## 6. Non-Surgical Treatment of IPF-LC Patients: Possible Options, Limitations, and Challenges

Some IPF-LC patients do not qualify for surgical treatment due to the advanced stage of cancer, poor lung function, and/or other comorbidities. Alternative oncological treatments include chemotherapy, radiotherapy, immunotherapy, and targeted therapies. Additionally, some procedures of oncological treatment gain increasing interest and significance in the therapy of ILD patients, such as percutaneous ablation of lung tumors or proton beam therapy (PBT). However, even those methods that are non-invasive pose a risk for patients with ILD. Patients with fibrotic ILDs are often disqualified from palliative therapy due to poor response to treatment and a high incidence of complications [181].

### 6.1. Percutaneous Ablation of Lung Tumors

Commonly, a number of IPF patients with early NSCLC stages are disqualified from LC resection surgeries due to insufficient lung function or comorbidities. A promising minimally invasive alternative for such patients is percutaneous image-guided thermal ablation (IGTA) of lung tumors. Unlike LC resection surgeries, IGTA preserves lung function, which is highly desirable in IPF patients. IGTA is a needle-directed procedure that utilizes high-temperature radiofrequency ablation (RFA) and microwave ablation (MWA) or cold-temperature cryoablation [181,182]. A retrospective study by Kwan et al. demonstrated that IGTA had similar efficiency and OS to sublobar resection in stage I NSCLC without ILD [181,183]. However, data on IGTA in ILD, particularly in IPF patients, remain limited. A recently published retrospective single-center cohort study by Kaseda et al. examined 37 ILD patients, including 15 patients with IPF who underwent a cryoablation procedure for LC. AE occurred in two patients and was the only cause of death within 60 days post-procedure. The mortality rate was 13.3% in IPF and 5.4% in all ILD patients [182,184]. Peng et al. retrospectively compared 27 IPF and 80 non-IPF patients with stage I NSCLC who underwent MWA of the LC. There were no post-procedure AE cases. The rates of adverse events were similar between the groups (48.6% IPF and 47.7% non-IPF, *p* = 0.998), although survival was worse in the IPF group. The primary cause of death was IPF-related rather than MWA-associated. Furthermore, there was no significant difference in progression-free survival (PFS) between groups [185].

IGTA is a promising treatment option for early-stage LC in patients who are not candidates for surgical resection. However, further research is needed to assess its safety and efficacy in ILD/IPF patients due to the currently limited data.

### 6.2. Radiotherapy for Patients with IPF-LC

Radiotherapy is one of the fundamental treatment methods for LC. It is commonly used in early-stage NSCLC for inoperable patients due to comorbidities or poor lung function. Additionally, radiotherapy can be applied in locally advanced NSCLC in combination with chemotherapy or as an adjunct to non-radical surgical treatment [50]. To date, no prospective studies have evaluated the impact of radiotherapy on ILD patients. This may be due to concerns about the risk of radiation pneumonitis (RP), a major complication of radiotherapy, which has led to the frequent exclusion of ILD patients from radiotherapy treatment. Retrospective studies showed that RP occurs more often in ILD than in non-ILD patients. An analysis of 537 patients with stage I NSCLC treated with stereotactic body radiotherapy (SBRT), including 39 ILD patients, revealed a significantly higher incidence of RP in ILD than in the non-ILD group (RP grade ≥ 2, 20.5% vs. 5.8%; *p* < 0.01, RP grade ≥ 3, 10.3% vs. 1.0%; *p* < 0.01) [186,187]. Onishi et al. reported a mortality of 6.9% of patients with RP related to SBRT in patients with pulmonary interstitial changes and stage I NSCLC [188].

Furthermore, the systematic review by Saha et al. showed that patients with IPF or UIP patterns were more likely to develop severe RP and had worse OS than other ILD patients [189]. KL-6 > 500 U/mL, serum surfactant protein D (SP-D), low FVC, and FEV1 were the independent risk factors for severe RP development. Furthermore, the volume of radiated normal lung parenchyma and the radiation dose were associated with more frequent RP occurrence [181,189].

As mentioned above, radiotherapy is an option for early-stage NSCLC in inoperable patients, with SBRT being the preferred method due to its lower RP rate compared to fractionated radiotherapy. In locally advanced NSCLC, where SBRT is insufficient due to a larger treatment field, fractionated radiotherapy is required. However, larger radiation fields and higher radiation doses increase the risk of RP. Furthermore, sequential radiochemotherapy is preferred over concurrent due to the lower risk of pneumonitis [181].

A retrospective study by Li et al. assessed patients with subclinical ILD and locally advanced NSCLC treated with fractionated radiotherapy and chemotherapy; 51.7% of them developed RP ≥ grade 2, including 5.7% with RP of grade 5 [181,190].

Given the higher risk of radiation-related complications in ILD patients, there is an urgent need to develop strategies to minimize radiation exposure. One promising approach is PBT, which theoretically reduces radiation exposure to healthy lung parenchyma. However, there is no consensus on whether PBT should be preferred over SBRT. A systematic review by Saha et al. reported a higher incidence of RP in ILD patients treated with PBT compared to SBRT, with median RP grade ≥ 2 rates of 43.87% vs. 32%, respectively [189]. In contrast, a study by Kim et al. on IPF patients with stage I-II NSCLC found a lower median incidence of RP in PBT-treated patients compared to SBRT-treated patients (12.5% vs. 40.9%), although the results were not statistically significant due to the smaller PBT group [191]. Further studies are needed to resolve these discrepancies and determine the optimal radiotherapy approach for ILD/IPF patients.

### 6.3. Chemotherapy for NSCLC

Chemotherapy plays a crucial role in LC treatment, either as part of palliative care or as an adjunct to radical therapy [50]. Although chemotherapy is also used in IPF-LC patients, their survival outcomes are generally poorer compared to non-IPF patients. A retrospective study by Kanaji et al. evaluated 218 patients with stage IIIB and stage IV NSCLC, including 53 patients with ILD, of whom 34 had IPF. These patients were treated with chemotherapy and/or molecular-targeted therapy, such as tyrosine kinase inhibitors (TKI). ILD patients had significantly worse outcomes than non-ILD patients, with shorter OS (267 days vs. 539 days), PFS (118 days vs. 196 days), lower disease control rate (DCR) (71% vs. 87%), and response rate (RR) (37% vs. 55%). Among ILD patients, those with IPF had even worse OS (233 days), PFS (92 days), DCR (53%), and RR (31%) [192]. The reduced effectiveness of chemotherapy in IPF-LC patients may be partly due to the lower incidence of *EGFR* mutations. This type of tumor is characterized by a poor response to therapy. Additionally, ILD and IPF patients experience adverse events more frequently during chemotherapy than non-ILD patients, often leading to early treatment discontinuation [192]. As with other LC treatment methods, ILD patients are also at risk of AE-ILD induced by chemotherapy. Studies report that the incidence of chemotherapy-related AE in ILD patients ranges from 5% to 20% [181]. A meta-analysis by Wang et al. identified several risk factors for chemotherapy-induced AE in ILD-LC patients, including age < 70 years, UIP pattern, low FVC, and elevated serum SP-D levels [193].

Implementation of standard-of-care systemic therapy for LC management in ILD patients is not always a good solution. Optimal systemic therapy regimens must balance efficacy and specific safety restrictions. Unfortunately, there is limited data on the safety of particular drug combinations and treatment regimens [181]. Some prospective studies examined these considerations for ILD-NSCLC patients. Two phase II trials by Kenmotsu et al. and Asahina et al. evaluated patients who received a combination of carboplatin and nanoparticle albumin-bound paclitaxel (nab-paclitaxel), where 4.3% and 5.6% of patients developed AEs, respectively [181,194,195]. In the phase II trial by Fukuizumi et al., carboplatin + weekly paclitaxel was associated with 12.1% of AEs [181,196]. Sekine et al. and Hanibuchi et al. studied carboplatin + oral fluoropyrimidine derivative S-1, reporting 9.5% and 6.1% incidences of AEs [181,197,198]. There are no prospective studies that assessed pemetrexed-based therapy (the most common regimen for NSCLC treatment) [181]. Existing data only come from small retrospective studies, indicating an AE incidence of approximately 10% [181,199,200].

#### 6.3.1. Chemotherapy for SCLC

SCLC has a distinct pathophysiology and treatment approach compared to NSCLC. Chemotherapy regimens for SCLC differ from those used in NSCLC, which may result in different impacts on IPF patients. The study by Watanabe et al. retrospectively enrolled 11 patients with IPF and extensive disease (ED) of SCLC. The RR was 63.3%, with a median PFS of 4.7 months and OS of 7 months. Four patients experienced chemotherapy-induced rapid deterioration, which was fatal in three cases [201]. Another study assessed 59 IPF-SCLC patients, including 30 who underwent chemotherapy and 21 who received chemoradiotherapy. The median PFS, OS, and RR for chemotherapy were 7.1 months, 9.9 months, and 64.3%, respectively. AEs occurred in nine patients (31%) after chemotherapy and in six patients (28.6%) after concurrent chemoradiotherapy [202].

In Koyama et al.’s study, the group of SCLC-IPF counted 20 patients and 27 with SCLC and idiopathic interstitial pneumonia (IIP) other than IPF. Chemotherapy-induced AEs occurred in 40% of IPF patients compared to only 3.7% of those with other forms of IIP. IPF was identified as an independent factor for AE development after chemotherapy (*p* = 0.007). However, there were no significant differences between IPF and other IIP groups in RR (60% vs. 72%) or DCR (85% vs. 88%) after the first line of chemotherapy. Despite this, IPF patients had significantly shorter OS than those with other IIPs (244 vs. 386 days, *p* < 0.001) [203].

The largest, relatively recent multicenter retrospective study from Japan included 492 IIP-NSCLC and 216 IIP-SCLC patients, with 406 IIP cases having an IPF diagnosis. The incidence of AEs following first-line chemotherapy was significantly higher in NSCLC than in SCLC (12.6% vs. 4.2%; OR: 3.316; 95% CI: 1.25–8.8). This suggests that chemotherapy regimens used for NSCLC may have greater potential to provoke AEs than those used for SCLC [204]. A recently published single-arm phase II study by Matsumoto et al. evaluated 31 IIP patients with advanced SCLC treated with carboplatin and etoposide. After a median of four chemotherapy cycles, no patients experienced AE. The RR was 83.9%, with a median PFS of 5.9 months and OS of 14 months [205]. SCLC remains an important factor influencing IPF patients’ outcomes. Given the differences in chemotherapy responses between NSCLC and SCLC, further research is essential to better understand treatment efficacy and safety in this population.

#### 6.3.2. Prevention of Chemotherapy-Induced AE

Emerging data suggest that antifibrotic therapy may help reduce the risk of AE induced by chemotherapy in patients with IPF. In a small-sample-size retrospective cohort study, out of 13 IPF-NSCLC patients who received doublet chemotherapy and pirfenidone, none developed AEs [181,206]. A pilot study by Makiguchi et al. enrolled 27 patients with IPF and advanced NSCLC who were treated with a combination of carboplatin, paclitaxel, and nintedanib. AEs occurred in four patients (14.8%), leading to the premature termination of the trial [207]. A phase III RCT (J-SONIC) enrolled 243 IPF patients with advanced chemotherapy-naïve NSCLC, who were treated with carboplatin plus nab-paclitaxel. The intervention in the experimental group was nintedanib administration (150 mg twice daily) and the primary end-point was exacerbation-free survival (EFS). The primary end-point of the study was not met. There was no significant difference in EFS between the two groups (median 14.6 months in the nintedanib plus chemotherapy group versus 11.8 months in the chemotherapy group (HR 0.89; 90% CI 0.67–1.17; *p* = 0.24)). There was no difference between groups in time to first AE. Notably, the benefits of nintedanib in terms of lung function, RR, and PFS were observed, as well as OS exclusively improved in patients with non-squamous histology of LC. There was no significant difference in quality of life between groups. Patients receiving nintedanib experienced adverse effects more frequently, including leukocytopenia, neutropenia, febrile neutropenia, elevated aminotransferase levels, proteinuria, and diarrhea. These adverse events were associated with a higher rate of treatment discontinuation or nintedanib dose reduction [208].

There is another drug that is considered to prevent chemotherapy-induced AE in ILD patients, which is the VEGFR inhibitor bevacizumab. In a retrospective study, patients with advanced ILD-NSCLC were treated with different chemotherapy regimens with bevacizumab or without. The incidences of chemotherapy-related AE-ILD were significantly lower in the bevacizumab than in the non-bevacizumab group (0% vs. 22.6%, *p* = 0.037) [199].

The current knowledge gap is even more pronounced in IPF-SCLC. In a recently conducted, single-arm multicenter phase II trial from Japan, 33 IPF-SCLC patients were treated with a combination of carboplatin, etoposide, and nintedanib. Notably, only one patient (3%) developed AE following the final chemotherapy administration [209].

Despite the lower RR and safety concerns compared to non-IPF patients, chemotherapy remains a viable treatment option for IPF patients with LC. The potential role of antifibrotics and bevacizumab in mitigating chemotherapy-induced AE is particularly promising and may enhance the safety profile of these regimens. Further research is essential to establish clear treatment guidelines and optimize chemotherapy strategies for this high-risk patient population.

### 6.4. Targeted Therapies and Immunotherapy

In addition to chemotherapy, which affects all cells in the body, targeted therapies such as EGFR and ALK inhibitors specifically target cells expressing certain molecular markers. One major complication of these therapies is pneumonitis. Recent meta-analyses indicated that NSCLC patients receiving ALK inhibitors developed pneumonitis in 2.14% of cases, while those treated with EGFR inhibitors experienced this complication in 5.2% of cases [50,181,210,211]. There is legitimate suspicion that patients with preexisting ILD experience pneumonitis more often than the general population.

In a prospective cohort study of 1482 Japanese patients with advanced or recurrent NSCLC treated with gefitinib, 4% developed pneumonitis. Among these, 27% had preexisting ILD, which was associated with a significantly increased risk of pneumonitis. The severity of ILD had an exact influence on the development of pneumonitis, with OR ranging from 4.8 in mild ILD to 25.3 in severe ILD compared to patients without ILD. Moreover, in the gefitinib group, the 12-week mortality rate for patients who developed pneumonitis was 58.7%, compared to 14.6% in those who did not [181,212]. Unfortunately, data on pneumonitis caused by targeted therapies in ILD-LC patients remain limited, highlighting the need for further research to better understand this phenomenon.

Therapy with immune checkpoint inhibitors (ICIs), which include PD-1 and PD-L1 inhibitors, represents a novel approach to systemic LC treatment [181,186]. However, a potentially life-threatening complication of ICI treatment is ICI-related pneumonitis. Notably, this condition must be differentiated from other entities that may occur during oncological treatment in IPF patients, such as infections, AE, RP, or TKI-induced pneumonitis [213]. Current evidence suggests that ILD-LC patients have approximately a 3 times greater risk of developing this condition [181,214,215,216]. In a retrospective study by Tasaka et al., 15 of 49 ILD-NSCLC patients treated with nivolumab or pembrolizumab developed ICI-related pneumonitis compared to 9.5% in the non-ILD group (*p* < 0.01) [216]. Additionally, in a similar study by Kanai et al., ILD patients were more likely to experience a severe course of ICI-related pneumonitis than those without ILD (19% vs. 5%, *p* = 0.022) [214]. A systematic review by Frank et al., analyzing nine retrospective studies, reported that the incidence of ICI-related pneumonitis in ILD-LC patients ranged from 7.3% to 42.9% [181]. Despite the increased risk, ILD should not be considered an absolute contraindication for immunotherapy. There is limited data on the mortality of ICI-related pneumonitis. In the few available studies, only isolated cases of death have been reported [181]. Although ILD patients experience a higher incidence of ICI-related pneumonitis, available data suggest that their RR, PFS, and OS are comparable to those of non-ILD patients [181,216]. However, given the limited data on ICI-related pneumonitis in ILD patients, further studies are necessary to better assess the risks and benefits of immunotherapy in this population.

## 7. The Promising Potential of Antifibrotics in Preventing LC Development in Patients with IPF

Nintedanib has been registered as a second-line treatment for NSCLC with ADC histology and also as an antifibrotic drug that slows down IPF progression; however, no prospective studies have evaluated its potential role in preventing LC development in IPF patients [217,218]. Unlike nintedanib, for which data on the prevention of LC development in IPF are lacking, some available data support such a role for pirfenidone in this context. A retrospective study by Miura et al. analyzed 296 IPF patients, including 118 who received pirfenidone. Notably, only 2.4% of IPF patients treated with pirfenidone developed LC, compared to 22% in those who did not receive the drug (*p* < 0.0001). Furthermore, multivariate Cox proportional hazards regression analysis demonstrated that pirfenidone significantly reduced the risk of LC development (hazard ratio [HR]: 0.11; 95% CI: 0.03–0.46; *p* = 0.003) over the study follow-up period [219].

Emerging evidence from two large, recently published retrospective cohort studies suggests potential anti-neoplastic effects of pirfenidone. The first study, conducted in South Korea, included 5038 IPF patients, 880 of whom were treated with pirfenidone. Unexpectedly, the incidence of LC was higher in the pirfenidone group than in the non-pirfenidone group (13.5% vs. 8.9%). This finding may be attributed to the fact that data were collected from a period when the inclusion criteria for antifibrotic drugs were strict, as antifibrotic therapy was only prescribed to patients with severe disease, who inherently have a higher risk of developing lung cancer. However, all-cause mortality among IPF-LC patients was significantly lower in the pirfenidone group compared to those who did not receive the drug (HR 0.61; 95% CI: 0.43–0.85) [136]. Another novel nationwide population-based cohort study in Korea retrospectively evaluated 9938 patients with an IPF diagnosis established between 2004 and 2019, 31.6% (3188) of whom were receiving pirfenidone. The median follow-up was 3 years, with a 1-year median time of pirfenidone treatment. A total of 766 patients developed LC, corresponding to rates of 6.7% in the pirfenidone group and 11.0% in the non-pirfenidone group. The LC incidence was 10.4 per 1000 person-years in the pirfenidone group, compared to 27.9 per 1000 person-years in the non-pirfenidone group. The cumulative incidence of lung cancer (LC) in the pirfenidone and non-pirfenidone groups was as follows: at 3 years, 3.0% (95% CI: 1.3–6.7%) vs. 7.5% (95% CI: 6.0–9.4%); at 5 years, 4.7% (95% CI: 2.8–8.0%) vs. 12.9% (95% CI: 11.2–14.9%), respectively (*p* = 0.012). Furthermore, propensity score analysis using inverse probability of treatment weighting (IPTW) was conducted to balance baseline covariates and enhance the validity of comparisons between groups. The results were consistent with those observed in the clinical cohort. Eventually, pirfenidone was independently associated with a reduced risk of LC (HR 0.347; 95% CI: 0.258–0.466). Although the results were consistent across all treatment durations, they reached greater significance with prolonged treatment, especially among those treated for two years or more [135].

It is essential to note that these findings are derived from retrospective studies, and their interpretation should be approached with caution due to the potential limitations and biases inherent in observational research. Retrospective analyses should not be considered confirmatory but rather hypothesis-generating. Therefore, prospective and randomized controlled trials are essential to determine whether antifibrotics can effectively prevent LC development in patients with IPF.

## 8. Conclusions

IPF significantly increases the risk of LC development, and the coexistence of both IPF and LC significantly worsens patients’ prognosis. Major risk factors for LC occurrence in patients with IPF include smoking, male sex, and older age. Numerous shared cellular, molecular, and epigenetic pathways between IPF and LC have been identified, potentially serving as novel targets for future drug development. A key challenge in the diagnosis and treatment of LC in IPF patients is the occurrence of AE and LC recurrence. The potential risks and benefits of various therapeutic approaches must be carefully weighed to determine the most appropriate clinical management strategy. An individualized, patient-centered approach is crucial for optimizing outcomes in patients with IPF-LC. Hence, establishing standardized guidelines is critical for optimizing treatment decisions and ensuring consistent, high-quality care.

## Figures and Tables

**Table 1:** Lung cancer incidence in patients with IPF and its basic characteristics based on recently published studies.

Author	Published Year	Reference	Number of Patients, n	IPF-LC n (%)	Median Time to LC Diagnosis (Months)	Men (%)	Age at IPF Diagnosis	Average Pack-Years
Ozawa	2009	[25]	103	21 (20)	120	95.23	NA	73.1
Lee	2012	[30]	1685	114 (6.8)	17	94.7	68.5	44.3
Hyldgaard	2014	[28]	121	7 (6)	NA	NA	NA	NA
Tomassetti	2015	[27]	181	23 (13)	30	82.6	66.9	36.7
Kato	2018	[29]	632	70 (11.1)	NA	94.3	66.8	46.4
Yoo	2019	[20]	938	135 (14.5)	38	94.8	69	41.7
Tzouvelekis	2020	[31]	1016	102 (10)	33	94.1	71.4	NA
Karampitsakos	2022	[24]	3178	324 (10.2)	28	90.4	71	NA
Lee	2023	[26]	21,111	706 (3.3)	NA	87.54	70.5	NA

Abbreviations: IPF—idiopathic pulmonary fibrosis; LC—lung cancer; NA—not available.

**Table 2:** The incidence of AE and mortality due to AE in patients with IPF undergoing LC resection.

Original Articles					
Author	Reference	Published Year	Number of Patients, n	Number of AEs, n (%)	Number of AE Deaths, n (%)
Kanazaki	[163]	2010	40	12 (30)	3 (25)
Park	[164]	2011	100	28 (28)	13 (46.7)
Maniwa	[165]	2013	89	8 (9)	1 (12.5)
Sato	[159]	2014	1763	164 (9.4)	72 (43.9)
Omori	[166]	2015	103	5 (4.90)	3 (60)
Joo	[167]	2016	80	6 (7.5)	NA
Kobayashi	[168]	2016	137	17 (12.4)	7(41.2)
Tsutani	[169]	2017	107	6 (5.6)	NA
Shao	[162]	2019	97	7(6.2)	5 (83.3)
Tang	[170]	2020	156	7 (4.5)	5 (71.4)
Kato	[171]	2023	68	8 (11.8)	1 (12.5)
Fujiwara	[172]	2023	91	14 (15.4)	9 (64)
Meta-analysis					
Hao	[160]	2022	2655	258 (9.7)	NA
Patel	[161]	2023	2202	322 (14.6)	NA

Abbreviations: AE—acute exacerbation; NA—not available.

## Data Availability

No new data was created for the purpose of this review.

## Abbreviations

ACEs	Alveolar ephitelium cells
ADC	Adenocarcinoma
AE	Acute exacerbation
AKT	Protein kinase B
ALK	Anaplastic lymphoma kinase
APOBEC	Apolipoprotein B mRNA editing enzyme, catalytic polypeptide
ASA	American Society of Anesthesiology
BAL	Bronchoalveolar lavage
BALf	Bronchoalveolar lavage fluid
BRAF	B-Raf proto-oncogene, serine/threonine kinase
BMI	Body mass index
CAF	Cancer-associated fibroblast
CSC	Cancer stem cells
CDKN1A	Cyclin-dependent kinase inhibitor 1A
CL13	C-C motif chemokine ligand 13
COPD	Chronic obstructive pulmonary disease
CRP	C-reactive protein
Cx43	Connexin 43
CXCL2	C-X-C motif chemokine ligand 2
CT	Computed tomography
DCR	Disease control rate
DLCO	Diffusing capacity of lungs for carbon monoxide
DNA	Deoxyribonucleic acid
ECM	Extracellular matrix
EGF	Epithelial growth factor
EGFR	Epithelial growth factor receptor
ED	Extensive disease
EFS	Exacerbation-free survival
EMT	Epithelial-to-mesenchymal cell transition
EndMT	Endothelial-to-mesenchymal cell transition
ER	Endoplasmatic reticulum
FAK	Focal adhesion kinase
FEV1	Forced expiratory volume in 1 s
FGF	Fibroblast growth factor
FHIT	Fragile histidine triad
GAP index	Genomic Age and Physiology index
GGO	Ground-glass opacity
HR	Hazard ratio
HRCT	High-resolution computed tomography
ICIs	Immune checkpoint inhibitors
ICU	Intensive care unit
IGTA	Image-guided thermal ablation
IIP	Idiopathic interstitial pneumonia
ILD	Interstitial lung disease
IL-1β	Interleukin 1 beta
IL-4	Interleukin 4
IL-5	Interleukin 5
IL-6	Interleukin 6
IL-12	Interleukin 12
KL-6	Krebs von den Lungen-6
KRAS	Kirsten rat sarcoma virus gene
LC	Lung cancer
Let-7d	Lethal-7d
LR-MSCs	Lung-resident mesenchymal stroma cells
MALT1	Mucosa-associated lymphoid tissue lymphoma translocation protein 1
MARCKS	Myristoylated alanine-rich C-kinase substrate
MGMT	O-6-methylguanine-DNA methyltransferase
miR-29	MicroRNA-29
miR-155	MicroRNA-155
miR-200	MicroRNA-200
miR-221	MicroRNA-221
miRNA	MicroRNA
MMP	Matrix metalloproteinase
MWA	Microwave ablation
NSCLC	Non-small-cell lung carcinoma
OR	Odds ratio
OSA	Obstructive sleep apnea LC
OS	Overall survival
PBT	Proton-beam therapy
PD-1	Programmed death-1
PD-L1	Programmed death-ligand 1
PDGF	Platelet-derived growth factor
PE	Pulmonary embolism
PET-CT	Positron emission tomography
PFS	Progression-free survival
PFT	Pulmonary function test
PH	Pulmonary hypertension
PI3K	Phosphoinositide 3-kinase
PLA2G7	Phospholipase A2 group VII
PF	Pulmonary fibrosis
RAS	Rat sarcoma virus
RFA	Radiofrequency ablation
RhoA	Ras homolog family member A
ROS	Reactive oxygen species
RP	Radiation pneumonitis
RR	Response rate
RTEL1	Regulator of telomere elongation helicase 1
SASP	Senescence-associated secretory phenotype
SBRT	Stereotactic body radiotherapy
SCC	Squamous cell carcinoma
SEMA6B	Semaphorin 6B
SFTPA1	Pulmonary surfactant-associated protein A1
SFTPB	Surfactant protein B
Shh	Sonic Hedgehog
SLB	Surgical lung biopsy
SMAD 4	SMAD family member 4
SCLC	Small-cell lung carcinoma
SP-D	Surfactant protein D
SPARC	Secreted protein acidic and cysteine-rich
SPP1	Secreted phosphoprotein 1
TBLC	Transbronchial lung cryobiopsy
TBLB	Transbronchial lung biopsy
TERC	Telomerase RNA component
TERT	Telomerase reverse transcriptase
TGF-α	Transforming growth factor alpha
TGF-β	Transforming growth factor beta
TKI	Tyrosine kinase inhibitor
TLA	Three-letter acronym
TLC	Total lung capacity
TLCD2	TLC domain containing 2
TNF-α	Tumor necrosis factor alfa
TP53	Tumor protein 53
UIP	Usual interstitial pneumonia
UPR	Unfolded protein response
VC	Vital capacity
%VC	Percentage of predicted vital capacity
VEGF	Vascular endothelial growth factor
VEGFR	Vascular endothelial growth factor receptor
Wnt/β-catenin	Wingless/Int beta-catenin
YAP	Yes-associated protein
